# Regions of open water and melting sea ice drive new particle formation in North East Greenland

**DOI:** 10.1038/s41598-018-24426-8

**Published:** 2018-04-17

**Authors:** M. Dall´Osto, C. Geels, D. C. S. Beddows, D. Boertmann, R. Lange, J. K. Nøjgaard, Roy. M. Harrison, R. Simo, H. Skov, A. Massling

**Affiliations:** 1 Institute of Marine Sciences (ICM) Consejo Superior de Investigaciones Científicas (CSIC), Pg. Marítim de la Barceloneta 37–49, 08003 Barcelona, Spain; 2 Arctic Research Centre, Department of Environmental Science, Aarhus University, 4000 Roskilde, Denmark; 3 Centre for Atmospheric Science Division of Environmental Health & Risk Management School of Geography, Earth & Environmental Sciences, University of Birmingham, Edgbaston, Birmingham, B15 2TT United Kingdom; 4 Department of Bioscience, Aarhus University, 4000 Roskilde, Denmark; 5 Department of Environmental Sciences/Center of Excellence in Environmental Studies, King Abdulaziz University, PO Box 80203, Jeddah, 21589 Saudi Arabia

## Abstract

Atmospheric new particle formation (NPF) and growth significantly influences the indirect aerosol-cloud effect within the polar climate system. In this work, the aerosol population is categorised via cluster analysis of aerosol number size distributions (9–915 nm, 65 bins) taken at Villum Research Station, Station Nord (VRS) in North Greenland during a 7 year record (2010–2016). Data are clustered at daily averaged resolution; in total, we classified six categories, five of which clearly describe the ultrafine aerosol population, one of which is linked to nucleation events (up to 39% during summer). Air mass trajectory analyses tie these frequent nucleation events to biogenic precursors released by open water and melting sea ice regions. NPF events in the studied regions seem not to be related to bird colonies from coastal zones. Our results show a negative correlation (r = −0.89) between NPF events and sea ice extent, suggesting the impact of ultrafine Arctic aerosols is likely to increase in the future, given the likely increased sea ice melting. Understanding the composition and the sources of Arctic aerosols requires further integrated studies with joint multi-component ocean-atmosphere observation and modelling.

## Introduction

Aerosols act as Cloud Condensation Nuclei (CCN’s), around which cloud droplets are formed^1^. The capability of aerosol particles to act as CCN has important implications for understanding the indirect aerosol-cloud effect^2^. Currently, atmospheric aerosols represent the largest source of uncertainty in global radiative forcing predictions^3^, especially in remote regions^4^. Within the Arctic, clouds are considered one of the most important factors for the surface energy balance^5^. The Arctic is a region particularly susceptible to climate change and it has warmed at a rate more than twice that of the global average since the mid-1960s^6^.

Different measurements at Arctic sites show a strong annual cycle in aerosol characteristics^7,8^. Due to the usually low concentrations of aerosol particles over the inner Arctic pack ice area in summer, natural surface particle sources have been emphasized to be much more important than transport from continental sources^9^. Primary ultrafine aerosols include biogenic micro-colloids shown to be polymer gels^10–12^, produced by phytoplankton and sea ice algae biological secretions. A number of studies have also reported *in situ* formation of new aerosol particles in the Arctic, which mostly involve new particle formation from natural emissions of volatile species that are oxidized in the Arctic boundary layer to low vapour pressure compounds^8,13^. The Arctic’s climate is a result of complex interactions between the cryosphere, atmosphere, ocean, and biosphere. Rapid sea ice loss is dramatically changing the Arctic surface^14^. Hence - to better understand the physical and chemical processes leading to a high nucleation/formation rate and a frequent appearance of clouds in the summertime Arctic - it is crucial to study the atmospheric natural emissions of the different surfaces in detail. Recently, Dall´Osto *et al*. (2017) linked NPF events detected at the Zeppelin site (Svalbard Islands) to open water and melting sea ice regions^15^. The formation and growth of these ultrafine particles seems to depend upon marine biological activities within the open leads and between the pack ice and/or along the Marginal Sea Ice zone (MIZ). It is important to stress that the NPF source regions and corresponding precursor components are still a topic of intense research, and not only include emissions of precursor gases associated with biological communities on or near sea ice margins^16,17^, but also seabird colonies^18,19^ and intertidal zones^20,21^. Freud *et al*.^22^ recently argued that there is no single site that can be considered as fully representative for the entire Arctic region with respect to aerosol number concentrations and distributions^22^. In this study, we aimed to understand how the retreat of the Arctic sea ice affects the formation of new particles at the Villum Research station, Station Nord (VRS) in north-eastern Greenland (81.6°N, 16.7°W, 24 m), which is 608 km to the west-northwest of Zeppelin - a more studied monitoring site^8,15^. VRS at Station Nord is a unique Arctic station located close to sea level at the ice stream from the Arctic Ocean. VRS is furthermore always located north of the polar Vortex representing the conditions of the high Arctic throughout the whole year.

## Results

### Categorising Arctic ultrafine aerosols and new particle formation events

**Figure 1:**
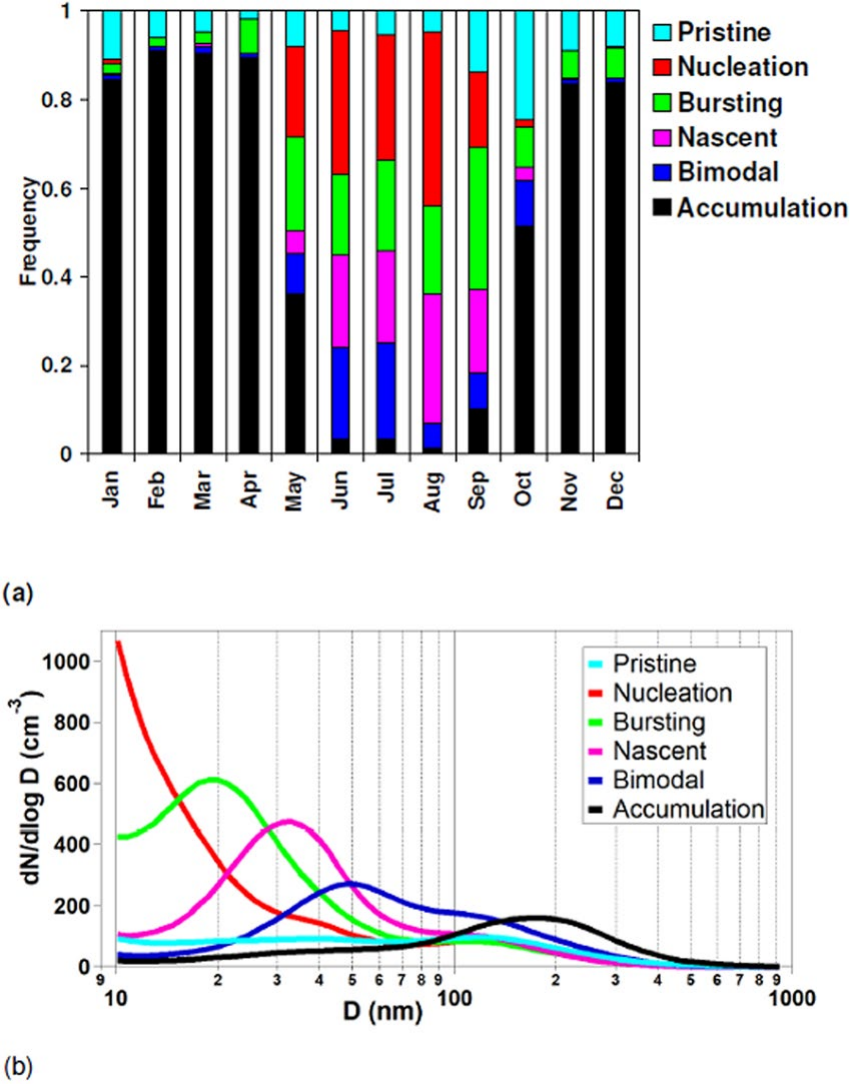
(**a**) Annual frequency distributions of the six aerosol categories; (**b**) average daily size distribution of the six aerosol categories.

K-means cluster analysis (see Methods) of particle number size distributions using 33,678 hourly distributions collected over 7 years (2010–2016, 55% data coverage during the period^13^) was carried out^23,24^. Based on such cluster analysis, we identified six categories of aerosol number size distributions. The annual seasonality is shown in Fig. 1a, whereas the corresponding average daily aerosol number size distributions are shown in Fig. 1b. Here, we refer to ultrafine as particles with diameters between 9 and 100 nm.

“*Pristine*” ultrafine. Occurring annually 10% of the time (min-max 2–24% based on monthly averages), this aerosol category is characterized by very low particle number concentrations (<100 particles cm^−3^). The minimum in aerosol number concentration is usually observed during September/October, confirming findings in previous studies in remote Arctic regions^8,13,22^. Figure 1b shows average aerosol number concentrations across different sizes, with two minor modes at 35 nm and 135 nm.

“*Nucleation*” ultrafine. Occurring annually 7% of the time, it presents a maximum in summer (July–August, 39%, Fig. 1a). Figure 1b shows the average daily aerosol number size distributions peaking in the smallest detectable size at 10 nm. The name of this category - which will be used below to represent new particle formation events - stands for continuous gas-to-particle growth occurring after the particle nucleation event. An example is shown in Figure S1 reporting a daily aerosol evolution starting in the morning at smallest-detectable sizes (9 nm) and reaching count mean diameters between 60–80 nm in the late afternoon (so called “banana-shaped plots”). Its diurnal profile peaks at 14:00–15:00; overall 95% of these events were detected during daylight months.

“*Bursting*” ultrafine. Occurring 9% of the time, this category shows a constant frequency during late spring and summer (17–21%, Fig. 1a) overall presenting a weaker seasonal variation relative to the *Nucleation* ultrafine category. Figure 1b shows the average number size distribution with an ultrafine mode peaking at about 20 nm. The name of this category refers to an aerosol population that bursts and begins to exist or develop, but fails to grow to larger sizes like in the nucleation category^15^. Whilst a fraction of these particles may be due to new particle formation with limited growth (so called “apple” new particle formation events^25^), or open ocean nucleation^26,27^, an Arctic ultrafine primary origin can also not be ruled out^9,28^.

“*Nascent*” ultrafine. This category occurs annually 8% of the time, with a strong seasonal trend peaking during summer (June-August, 21–29%) and with a broad Aitken mode centred at about 30 nm without showing a clear diurnal pattern (Fig. 1a,b). The name of this category emerges from growing ultrafine aerosol particles resulting from an array of different primary and secondary aerosol processes linked to emissions of local and regional marine origin^8^.

“*Bimodal*” ultrafine. Occurring annually 9% of the time, this category mainly occurs during the period May-October with a peak during June and July (21–22%). This aerosol category is characterized by a bimodal number size distribution peaking at about 50 nm and 135 nm in diameter (Fig. 1b). The standard picture of marine aerosols has been of a multi-modal distribution with a fine (Aitken) mode in the size range between 20 to 100 nm and an accumulation mode between 100 and 500 nm in diameter^29,30^.

“*Accumulation*”. This aerosol category is characterized by larger size modes that contribute only very little to the overall ultrafine aerosol population numbers. It was found to be the most frequent category occurring 56% of the time throughout the whole measurement period. From the month of November the number concentration of the accumulation mode aerosol slowly rises towards the next-year maximum that occurs during the following spring haze period (Fig. 1a, February-April, 89–91%)^8,31^.

In summary, our method allows apportionment of the Arctic aerosol complexity observed at Villum Research Station, Station Nord (VRS) in North Greenland, and to pinpoint six aerosol categories describing the whole aerosol population. In Figure S2, our current classification is compared with the previous similar study conducted at the Zeppelin (Svalbard) monitoring site^15^. Broadly, all *Accumulation* categories peak in size ranges above 100 nm. By contrast, the aerosol categories peaking in the smallest size ranges are the *Bursting* and the *Nucleation* ones. It is important to stress that NPF events at daily resolution are attributed to the *Nucleation* category, whereas the *Bursting* ones are defined by aerosols of about 10–30 nm not seen in the typical banana-shaped plots. Categories *Nascent* and *Bimodal* generally have a main Aitken mode (about 30–100 nm), resulting from the processing of local and regional marine aerosols. In the following sections, special emphasis is given to the five ultrafine aerosol categories, in particular to attemp to better understand the origin and driving sources of NPF precursors in the studied Arctic area.

### Association of NPF events with chemical and physical parameters

It is still debated as to what extent different natural and anthropogenic source regions contribute to the Arctic aerosol. The dominating transport of the accumulation aerosol category lies within an approximate 120-degree sector extending to Alaska in the easterly direction and northern Siberia in the westerly direction. Transport from land-based sources seems to be dominated by source regions from Siberia, Eurasia and to some degree the European subcontinent as reported in previous findings^32,33^. By contrast, during the summer months (June–August) a much larger fraction of air mass transport takes place over the Atlantic Ocean^34^. Nevertheless, black carbon measurements were conducted during the period 2011–2013 by a MAAP instrument^35^ in the same location. By investigating the relation between aerosol categories and black carbon concentration (Figure S3), we demonstrate clean conditions with very minor anthropogenic influence for categories *Pristine*, *Nucleation* and *Bursting* (average 9 ± 3 ng m^−3^, Figure S3). The remaining ultrafine categories (*Nascent* and *Bimodal*) show higher although still generally low concentrations (15–19 ng m^−3^, Figure S3). By contrast, aerosol category *Accumulation* is associated with the highest black carbon concentrations (55 ng m^−3^, Figure S3). The detailed chemical composition of the categorized Arctic ultrafine aerosol is not known at this stage as instruments with sufficiently low detection limits are not yet available. However, the black carbon concentrations associated with the different categories point to natural sources responsible for high ultrafine aerosol concentrations detected during summer months at this high Arctic site.

The condensation sink (CS) is a very important physical factor in influencing the NPF process. Homogeneous nucleation is unlikely to occur in environments with a high condensation sink as under such conditions, condensable molecules and clusters are likely to attach to existing surfaces rather than self-nucleating to form new particles. We calculated the condensation sink (Methods) for each aerosol category. As expected, the *Pristine* Arctic category showed the lowest average values (6.4 × 10^−4^ s^−1^, Figure S4a), followed by categories *Nucleation* (8.9 × 10^−4^ s^−1^) and *Bursting* (1.0 × 10^−3^ s^−1^). Other ultrafine categories showed values above about 1.1 × 10^−3^ s^−1^ (*Nascent*, *Bimodal*). When investigating the diurnal profile of the condensation sink for the ultrafine categories (Figure S4b), values for the *Pristine* category are constant over the day at around 0.6 × 10^−3^ s^−1^. In contrast, the category *Nucleation* shows the lowest CS only in the morning hours, whereas the CS increases during the day while newly formed particles increase in size, reaching values of about 1.1 × 10^−3^ s^−1^ in the late afternoon. A number of important conclusions can be drawn from Figure S4b. First: given similar low CS values during the beginning of NPF events (morning hours) for categories *Pristine* and *Nucleation*, the CS may not be a factor that directly limits the NPF in this region; supporting the study of Collins *et al*. (2017) stressing that summertime Arctic nucleation events are more common and widespread than in other remote sites^36^. Second: nucleating and/or growing particles occurring mainly in the afternoon hours can be an important contributor to total CS during summer and thus be a limiting factor for the initiation of additional new particle formation events. This is because CS values in the afternoon reach values comparable to other aerosol categories with high CS where nucleation events are not seen, likely because of unfavourable conditions. Third: the low CS values calculated for the *Pristine* category and the absence of nucleation events during these days suggest that the lack of gaseous precursors is the determinant for absence of nucleation events.

### Elucidating source regions

**Figure 2:**
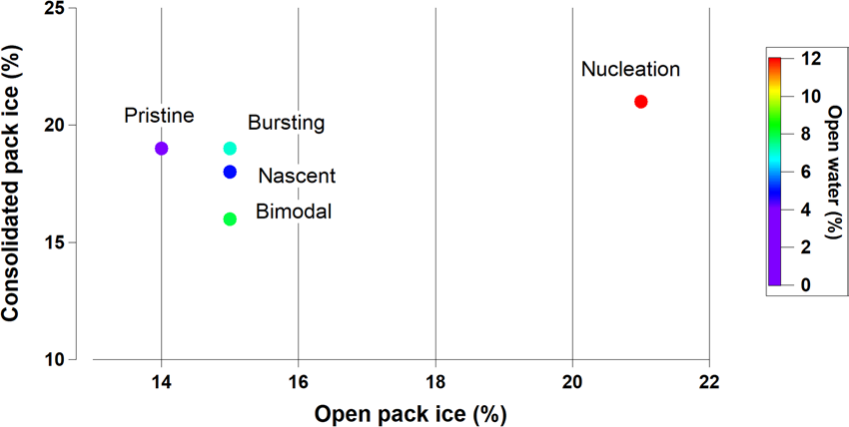
Percentages of total time (hours) of air mass back trajectories travelling over different sea ice areas for each of the five ultrafine aerosol categories.

There is an increasing number of studies using different approaches to identify the source regions of the major Arctic short-lived pollutants and their seasonality^22,36,37^. Broadly, they associate periods with high levels of anthropogenic pollutants to transport from northern Eurasia to the Arctic sites mainly during winter and spring. During summer the transport into the Arctic from mid-latitudes is minor due to the polar dome, and efficient removal processes may also play a role^7,22^. Therefore, short-lived pollutants are emitted locally and particles are formed *in situ* via gas to particle conversion processes^15^. We calculated about 10,300 air mass back trajectories aiming to shed some light on possible different source regions. Figure S5 shows the clustering results (see *methods*) of the air mass back trajectories, showing six main categories. Air masses mainly arrived from Greenland (cluster C1, C3), from the North East marine sector (C4, C6) and from marine and coastal Arctic zones (C2, C5), Unfortunately, no robust differences were found among the aerosol categories (Table S1). In a further analysis, we obtained information on how far each air mass travelled (total travel time 24, 60 and 120 h) over zones distinguished by their surface characteristics, namely land only, land covered by snow, sea ice and open water for each one of the different aerosol categories presented (see *methods*). Table S2 shows that category *Nucleation* is the one most associated with open water and sea ice (55% in total), supporting previous studies at Zeppelin Mountain^15^. Both open water (12%) and sea ice (43%) were found the highest percentages among different aerosol categories (Table S2). In our definition of sea ice regions, we classified “consolidated pack ice” as regions with pack ice concentration higher than 85%, “open pack ice” as regions with sea ice concentration higher than 15% and lower than 85% within the consolidated ice region, and “open water” as regions with sea ice concentrations lower than 15%^39^. For each day of each aerosol category, we calculated the amount of time spent by the associated air mass trajectory over the sea ice regions. Results are summarised in Fig. 2: the category *Nucleation* is the one mostly associated with open pack ice (21% in total, about 65% higher than the other four ultrafine aerosol categories). Given arctic new particle formation has been shown to be characterize by growth rates of the order of 0.01–15 nm per hour^36,38^, Table S2 provide the analysis for a range of air mass back trajectories (1-day, 3-day, 5-day), all supporting the fact that open water and melting sea ice regions are likely a biogenic source of new particle formation events. The current results are also highly consistent with a recent study carried out at the Arctic station Zeppelin Mountain^15^.

For the first time, we carried out additional analysis by taking melt pond information retrieved from Medium Resolution Imaging Spectrometer (MERIS) satellite data and overlapped this information with air mass back trajectory and aerosol cluster analysis^39,40^. Thin first-year sea ice is not structurally stable and prone to fracture and likely to result in the formation of leads (transient areas of open water surrounded by sea ice). Ice melting starts with the formation of visible pools, referred as to melt ponds, collecting melt water. Melt ponds penetrate the ice flow and represent an extending new habitat for sea ice microorganisms. Most of the Arctic surface area is covered by thin first year ice, forming at the beginning of fall to melt in late spring. In the Arctic summer, melt ponds commonly occur on Arctic sea ice and cover about 5–50% of the sea ice area^41–43^. In a nutshell, melt ponds can be defined as an accumulation of melt water on sea ice, mainly due to melting snow, but in the more advanced stages also due to the melting of sea ice. Results are plotted in Figure S6, where as expected the highest fraction of air masses travelled over melt pond areas is associated with the *Nucleation* category. However, the overall fraction of air mass travel time over melt ponds areas (<3% of the total) is much smaller relative to other areas (open water >12%, open pack ice >21%, Figure S6).

**Figure 3:**
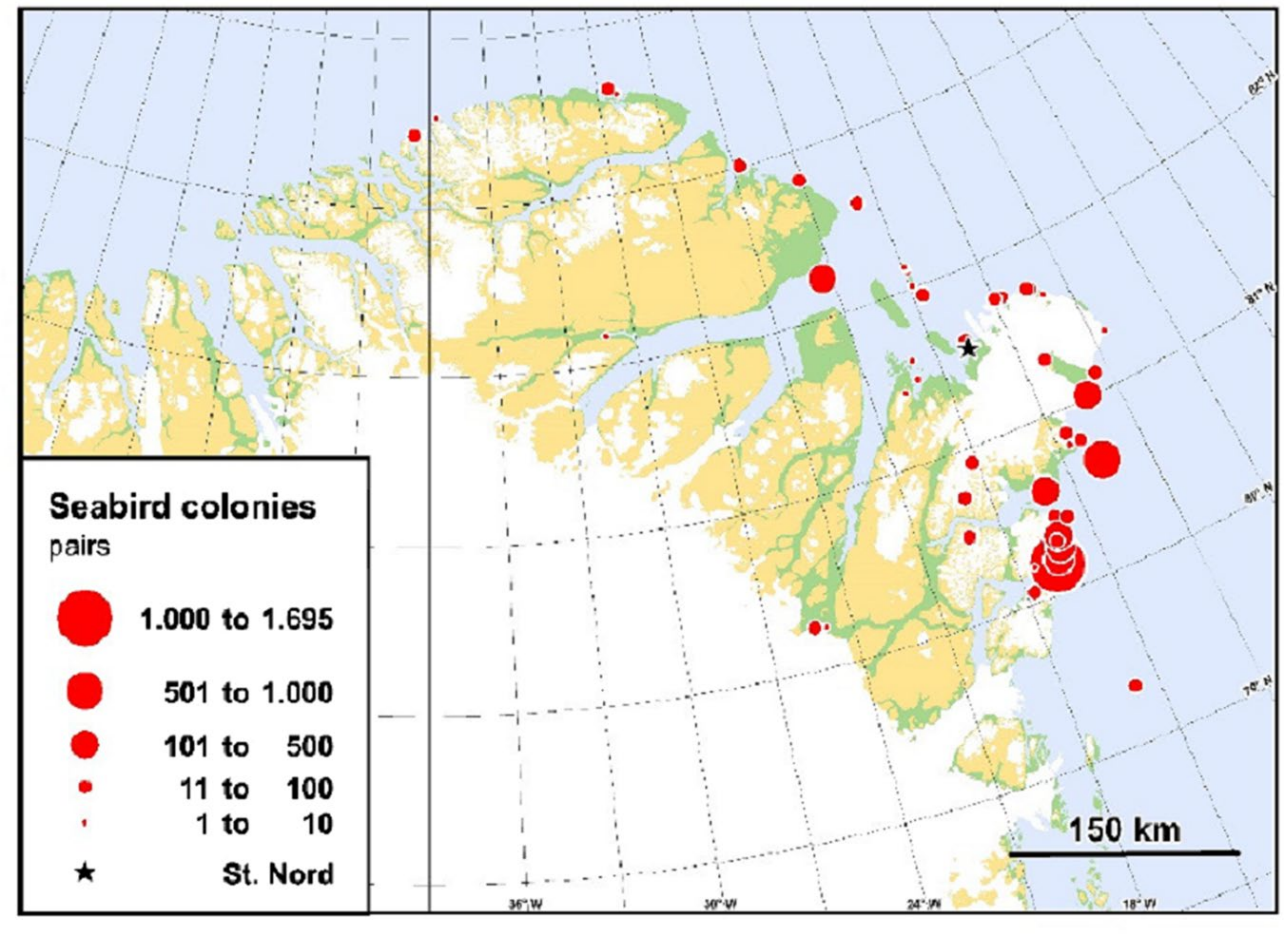
Map showing the distribution and size of seabird breeding colonies (n = 50) in the surroundings of Station Nord. All species are combined in the map. The species are: northern fulmar (Fulmarus glacialis), glaucous gull (Larus hyperboreus), black guillemot (Cepphus grylle), Sabines gull (Larus sabinii), ivory gull (Pagophila eburnea), black legged kittiwake (Rissa tridactyla) and Arctic tern (Sterna paradisaea). In total, approx. 4800 pairs of these seabirds are estimated to breed in the region. Land areas marked with green are land below 200 m asl, other areas are land above 200 m asl. White areas indicate inland ice. This plot was created using the R software (R Core Team (2016) R: A Language and Environment for Statistical Computing. R Foundation for Statistical Computing, Vienna, Austria. https://www.R-project.org/).

Finally - whilst sea ice seems to play a role in NPF events - it should not be forgotten that nucleation events were also associated with air masses that travelled over snow regions (Table S2), supporting a previously proposed mechanism based on gas precursors released from the snowpack^38^. Additional fundamental studies of biologically modified ocean-atmosphere interactions are needed in order to fully elucidate NPF formation and growth occurring in the Arctic.

### Possible contribution from seabird-colony guano to newly formed particles

The current understanding on mechanisms of new particle formation in the marine boundary layer over the Arctic Ocean is unclear due to the low concentration of nucleating agents^15,18,21,36^. Earlier work shows that seabird colonies represent a potential important source of ammonia to the atmosphere, especially in remote areas like the Arctic, where the anthropogenic contribution is low^18,44–46^. Croft and colleagues recently updated a global map of ammonia emissions from seabird-colonies by adding missing seabird colonies reported in the Circumpolar Seabird Data Portal for the region north of 50°N^45^. Based on information on these colonies they estimated the related ammonia emission by using the same bioenergetics model as in the global estimate. Here we take this a step further and focus on the region surrounding Station North in the most northeasterly part of Greenland. By extracting data from the Greenland Seabird Colony Register, a total of 50 seabird-breeding colonies were identified in the region. A map showing the distribution and size (number of pairs) of the colonies in the surroundings of Station Nord is given in Fig. 3. The included species are: northern fulmar (Fulmarus glacialis), glaucous gull (Larus hyperboreus), black guillemot (Cepphus grylle), sabines gull (Larus sabinii), ivory gull (Pagophila eburnea), black legged kittiwake (Rissa tridactyla) and Arctic tern (Sterna paradisaea). In total, approx. 4800 pairs of these seabirds are estimated to breed in the region around Station Nord. A few colonies with common eider have been excluded here, to be consistent with the original maps.

**Figure 4:**
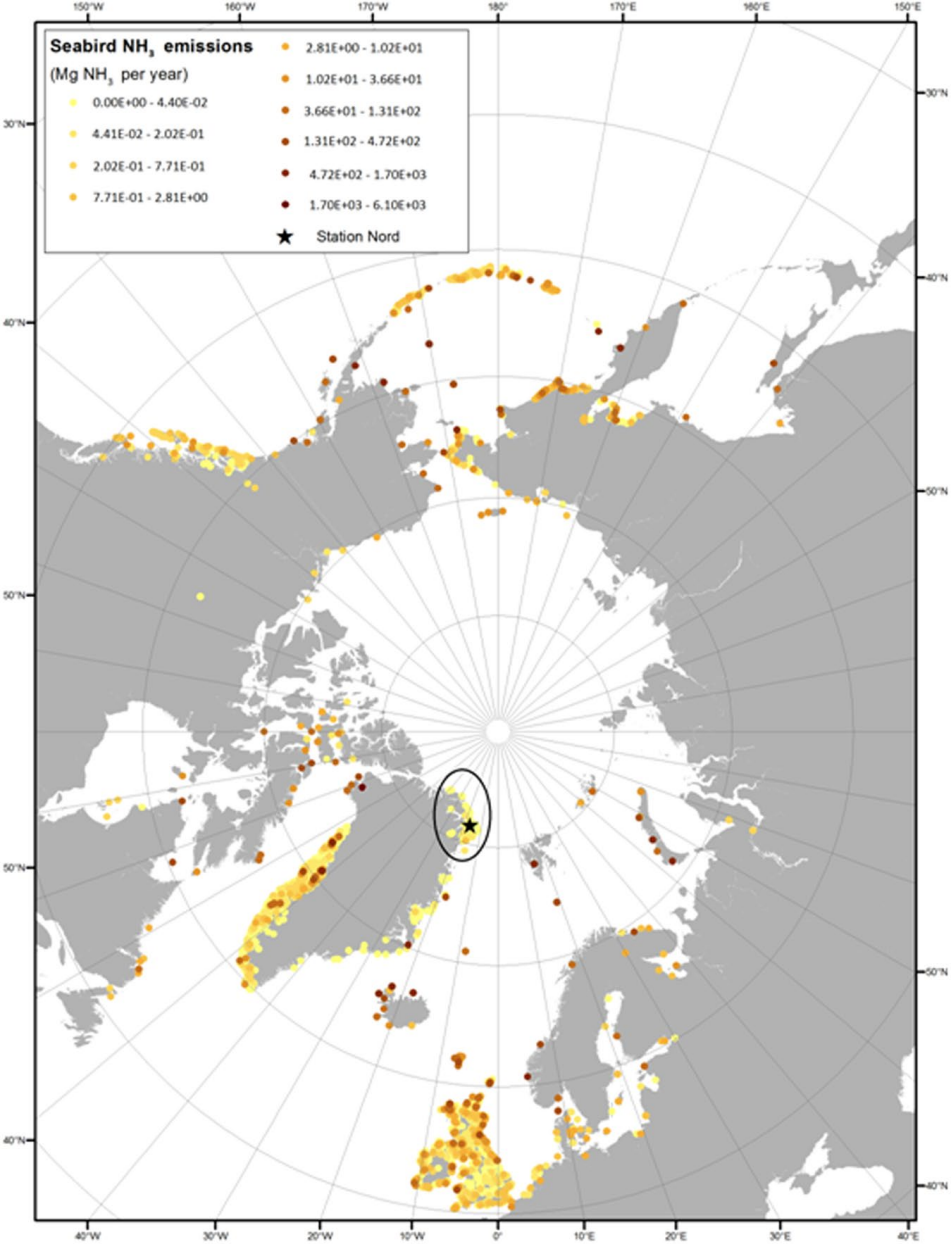
Updated map of annual NH_3_ emissions from seabird-colonies north of 50 N by combining this study with previous ones^18,46,47^. The emissions around Station Nord are estimated based on a bioenergetics model^48^ and the information on seabird-colonies from Fig. 3. Here the standard “mid-latitude” model is used for the Station Nord area. If the model parameters were adjusted towards lower temperatures and hence a lower volatilization of NH_3_, the emissions in this area would be even lower. Note that the legend spans over several orders of magnitude. This plot was created using the R software (R Core Team (2016) R: A Language and Environment for Statistical Computing. R Foundation for Statistical Computing, Vienna, Austria. https://www.R-project.org/).

We then used the same bioenergetics model as the previous studies^47^ to estimate species-specific annual ammonia emissions. Based on population and standardized bird specific information (e.g. food consumption, nesting habitat and the link to fraction of excreted nitrogen being volatilized) the model gives an estimate of the emission from seabirds (breeders, non-breeders and chicks). It is well known that the volatilization of ammonia is very temperature sensitive leading to significant impacts on the emission from agriculture^48,49^. Riddick *et al*. (2012)^45^ discussed this and found that for the yearly emission from seabirds in the Arctic area is about a factor of two higher when using the standard “mid-latitude” model compared to when adjusting for lower volatilization in a colder climate^45^. Using the standard model setup the total yearly emission from the colonies in Fig. 3. is estimated to be 2.31 Mg NH_3_ year^−1^. If the parameters are adjusted to a colder climate, the emission is only 0.69 Mg NH_3_ year^−1^. The previous studies assumed that the ammonia is emitted during a typical nesting period from 15th of May to 15th of September, but in this area the typical nesting period is from 1st of June to 15th of August. Based on the current knowledge it has not been possible to include short-term variation in the emission due to variations in meteorological drivers like temperature, precipitation and wind. Our results are therefore associated with a high uncertainty. The numbers of breeding seabirds in the region of Station Nord in northeast Greenland are very low compared to the numbers found along the northwest coast of Greenland. The main reason is that polar drift ice blocks most of the coasts and prevents access to open waters even in summer. There are open waters in spring/summer in the polynya – the Northeast Water (NEW) – and here the highest numbers of breeding seabirds are found (Fig. 3). However, the numbers there are still few compared to northwest Greenland, and this must be ascribed to the relatively low primary production combined with a strong benthic-pelagic coupling in this polynya^50–52^. The ammonia emission from birds in the region around Station Nord (Fig. 4) is therefore estimated to be much lower than along the western coast of Greenland and the Canadian part of the Arctic^18,36^. In a nuthshell, it is believed that - in the study area - bird colonies are not an important factor driving NPF events, mainly due to their small emissions. However, it is not excluded that NPF events in other parts of the Arctic could also contribute to particle number in some of the categories other than nucleation (e.g. bursting and nascent) because there could be transport of the growing particles from these other regions of NPF to Station Nord.

### Discussion and implications for climate

**Figure 5:**
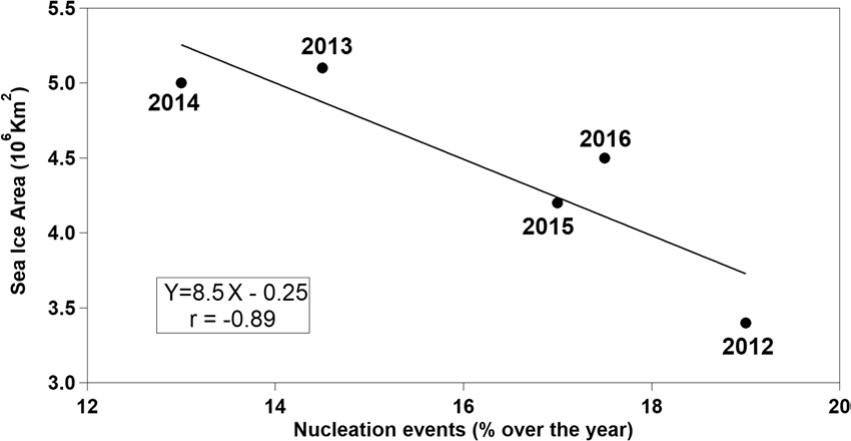
Relationship of sea ice extent (calculated over nearby geographic sectors of Greenland Sea, Barent Sea and Baffin Bay)^53,54^ with the temporal occurrence of the *Nucleation* aerosol category only.

The Arctic is an environment with low CCN concentrations, therefore the sensitivity of cloud droplet number concentrations and corresponding albedo changes to changes in CCN are typically stronger than in cases with larger CCN concentrations^1,34^. Our statistical analysis on seven years of aerosol number size distributions at Villum Research Station, Station Nord shows that ultrafine particles are more abundant than accumulation mode particles during summer in the boundary layer. Such ultrafine particles - likely arising from marine biogenic precursors - would require greater water vapour supersaturations to nucleate cloud droplets than accumulation mode particles. Large-scale atmospheric and oceanic phenomena as well as persistent weather patterns might affect the intra-Arctic as well as year-to-year variability of Arctic NPF. Following the study of Dall´Osto *et al*.^15^, we took nucleation events detected over the period 2010–2016 and compared it with the sea ice extent over the study area^15,53,54^. Five years of continuous measurements with data cover >85% are used in the analysis. Figure 5 shows a very good correlation (r = −0.89), suggesting that if the sea ice pack continues to retreat in the near future, increased secondary new particles will occur in the Arctic. The possibility that eventually the CS may be sufficiently increased by both the NPF events and increased primary emissions from open water regions - factors limiting formation of new particles - are not yet apparent in the analysis shown in Fig. 5.

The results herein presented from the period 2010–2016 at Villum Research Station, Station Nord are in line with recent results from the period 2000–2010 at the Zepplin Mountain Station^15^. Both measurement studies seem to disagree with modelling results reporting that increased summertime marine biogenic emissions will not cause a strong climate feedback due to the efficient removal processes for aerosols^55^. However, our level of understanding in the polar atmosphere lags behind the terrestrial biosphere to a great degree. The link between ocean biology and atmospheric processes is one of the most intriguing open questions in climate science. Lower sea ice surface concentrations combined with differences in the development stage and activity of marine microbial communities are observed to correlate with secondary NPF events^15,36^. The current study reports findings supporting this statement.

However, there is still uncertainty regarding the mechanism of aerosol production in the Arctic, especially from leads and open pack ice. Ecosystem interactions within the surface layer of the ocean are more important to air-sea chemical interactions compared to the state of any single biological variable. The production of sea ice exopolymers is not always coupled to algal growing seasons, adding complexity in detecting primary marine aerosol components. Other mechanisms may be also responsible for primary emissions, including the transport of bubbles to the surface by gases released from melting ice^9,56^ and a surface heat flux-driven mechanism^57^. Whilst Arctic secondary NPF events are being reported, the gas-phase precursors and reactive species are key remaining factors for explaining inter-annual differences and regional variability. Within the Arctic, the ratio of coastline to open waters can be relatively large compared to other regions^36^ enhancing the importance of volatile precursor sources at land-ocean boundaries like seabird colonies^18,19^ and intertidal zones^20,21^. New ice formation leads to enrichment of halogenides at the sea ice surface that can be readily oxidized to the highly volatile halogens (Cl_2_, Br_2_ and I_2_ or a combination of them e.g. ClBr)^58^, this might be the main source for IO_3_^−^ recently found as the major compound for particle formation during spring^21^. Other aerosol production mechanisms are also being proposed. A novel source of Oxidised Volatile Organic Compounds (OVOCs) to the marine boundary layer via chemistry at the sea surface microlayer was found on Arctic glass sea state associated with an organic enriched sea surface microlayer and low wind speeds^59^. These OVOCs do not correlate with levels of isoprene, monoterpenes or dimethyl sulphide supporting a different mechanism for OVOC emissions.

Our results highlight the importance of conducting continuous, long-term and high-resolution aerosol measurements at multiple high Arctic locations in order to characterize the aerosols across the Arctic throughout the year^22^. Our measurements highlight the importance of natural, marine inner-Arctic sources for summertime Arctic aerosol.

## Methods

### Location

The station represents remote Arctic conditions. Aerosol particles and trace gases were measured at the measurement site “Flyger’s Hut”, Villum Research Station, Station Nord (VRS), in northeast Greenland (81° 36′N, 16°40′W; 24 ma.s.l. The station is surrounded by multiyear sea ice, with limited bare ground occasionally and limited first-year ice along the coast of Greenland during the summer months.

At VRS polar sunrise is at the end of February, while polar day prevails from mid-April to the beginning of September and polar night prevails from mid- October.

### Aerosol size distribution

Detailed information SMPS can be found elsewhere. Measurement of particle number size distributions at Station Nord was initiated in July 2010 using a TROPOS-type Mobility Particle Size Spectrometer as described in Wiedensohler *et al*.^60^.

### Aerosol size distribution cluster

In order to group together the number size distributions (NSDs) into common sets which were dependent mainly on the shape of the distribution and not the magnitude, the NSDs were normalised to the vector sum and cluster analysed using k-means clustering^50^. K-means clustering aims to partition the observations into k clusters in which each observation belongs to the cluster with the nearest mean. The analysis works given a predefined number of clusters k and an optimum needs to be decided upon. The optimum cluster number was derived using the Dunn Index and Sihouette Width (silwidth). The Dunn index (DI) is a function of the ratio of the minimum cluster separation to the maximum cluster, implying that the larger the Dunn index the more compact and well separated. High values of DI identify sets of clusters that are compact, with a small variance between members of the cluster, and well separated, where the means of different clusters are sufficiently far apart, when compared to the within-cluster variance^51,52^. However, as the number of clusters increases there is a tendency of DI to decrease. A second useful measurement is the Silhouette width, which is a measure of the similarity of the SMPS spectra within a cluster (cohesion) compared to other clusters (separation). The range of the silwidth is from 1 to −1, where 1 indicates that the elements within the cluster are have a high similarity with each other but a low similarity with the elements with the other clusters. For the SMPS data there is a tendency for the silwidth to be high for smaller cluster numbers which decreases as the cluster number is increased as the separation of clusters decreases. There will be a cluster number at which the natural clusters start to divide. This is point is judged as the cluster number where a common abrupt change in the DI and silwidth takes place, i.e. at 8 clusters for the clustered daily SMPS spectra (silwidth = 0.43 and Dunn Index = 0.4.8 × 10^−3^). In practice, it is often the beneficial to select a higher number of clusters and then merge the clusters together manually by matching the size distributions and time series.

### Air mass back-trajectory

A back trajectory for each NSD cluster was calculated by averaging all the air mass back trajectories calculated with arrival dates corresponding to a measurement day of each average daily SMPS spectrum within each *k*-Mean NSD cluster. Using HYSPLIT4 (with revision made in February 2016), five day back trajectories were calculated for Station Nord from 2010 to 2016 using arrival hours of 00:00, 06:00, 12:00 and 18:00 and an arrival height of 10 m. In addition to the average back trajectory for each SMPS cluster, we had the addition facility of clustering the trajectories themselves using the ‘trajCluster’ function within the Cran R Package Openair^61^. Size trajectory clusters were chosen as best representing the dataset when using the ‘Euclid’ method within the cluster function (Figure S4).

### Melt ponds satellite data

Daily Polar Stereographic maps of the Northern Hemisphere classified each of 1024 × 1024 24 km grid cells as land, sea, ice or snow ice, and from this, the percentage of time each clustered back trajectory spent over each type could be calculated. The snow and ice coverage values were produced by the NOAA/NESDIS Interactive Multisensor Snow and Ice Mapping System (IMS) developed under the direction of the Interactive Processing Branch (IPB) of the Satellite Services Division (SSD)^62^. Regions are classified as: land, snow on land, sea ice and open water^62^. A similar calculation was repeated but using daily maps of sea ice and melt pond percentage concentration measured on a 12.5 km grid. These Artic polar sterographic maps of 12.5 km resolution contained sea ice concentration from the 85 GHz channel of SSM/I on DMSP, available since 1992. The percentages assigned from these maps to each trajectory step allow a ‘spectrum’ of sea ice concentration of 5% width from 0 to 100% to be calculated for each of the trajectory clusters. MODIS Arctic melt pond cover fractions (v02) were obtained for [2010–2016] from the Integrated Climate Data Center (ICDC, icdc.cen.uni-hamburg.de/), University of Hamburg, Hamburg, Germany, [Mar, 2017] and further details can be obtained from Rösel *et al*.^62^ and DOI:10.1594/WDCC/MODIS__Arctic__MPF_V02.

### Aerosol chemical tracers

Black carbon data taken at hourly resolution for the years 2012 and 2013 were considered in this analysis, further information can be found elsewhere^35^.

### Calculation of the Condensation Sink

The condensation sink (CS) describes how rapidly condensable vapour molecules will condense on the existing aerosol. Specifically this quantity describes the loss rate of molecules with diameter dp, diffusion coefficient D, and mean free path λv onto a distribution n(dp) (or Ni in the discrete case) of existing particles and as such, can be obtained from integrating over the particle size spectrum^63^. Calculation are described elsewhere^64^.

### Data availability

The data that support the findings of this study are available from the corresponding author on request.

## Electronic supplementary material

supplementary information
