# Supplementary material for: Regions of open water and melting sea ice drive new particle formation in North East Greenland
Source: Sci Rep. 2018 Apr 17;8:6109. doi: 10.1038/s41598-018-24426-8 (PMC5904185; doi:10.1038/s41598-018-24426-8)
Supplement: Supplementary file 1 — supplementary information [file 41598_2018_24426_MOESM1_ESM.pdf]

SUPPORTING INFORMATION

Regions of open water and melting sea ice drive new particle formation in North East Greenland

M. Dall´Osto, C. Geels, D. C. S. Beddows, D. Boertmann, R. Lange, J. K. Nøjgaard, Roy. M. Harrison, R. Simo, H. Skov and A. Massling

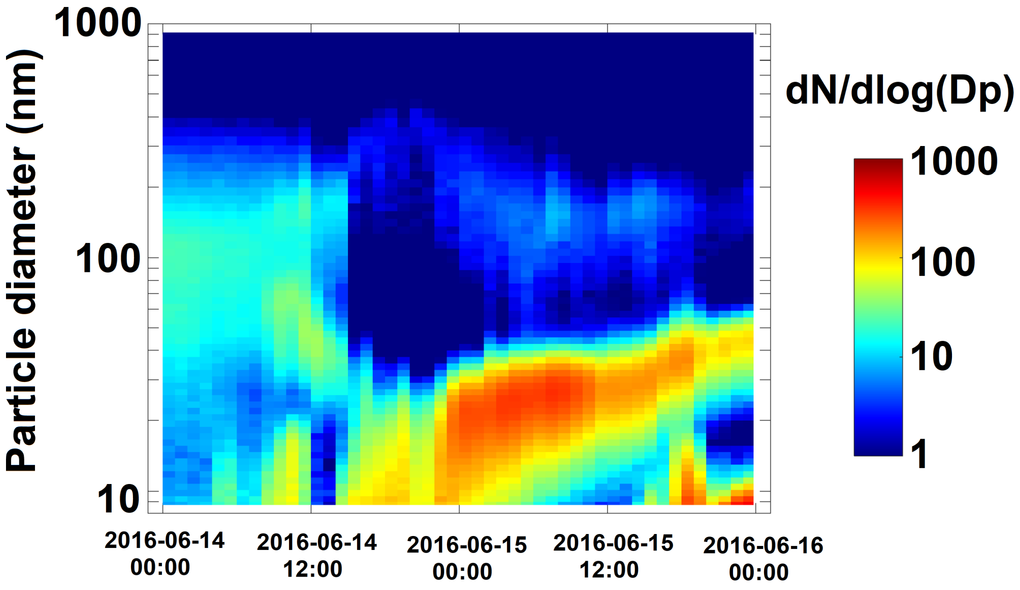

Supplementary Figure S1 Example of new particle formation event (Day 14th June 2017). These events are also called "banana-shaped plots"[8].

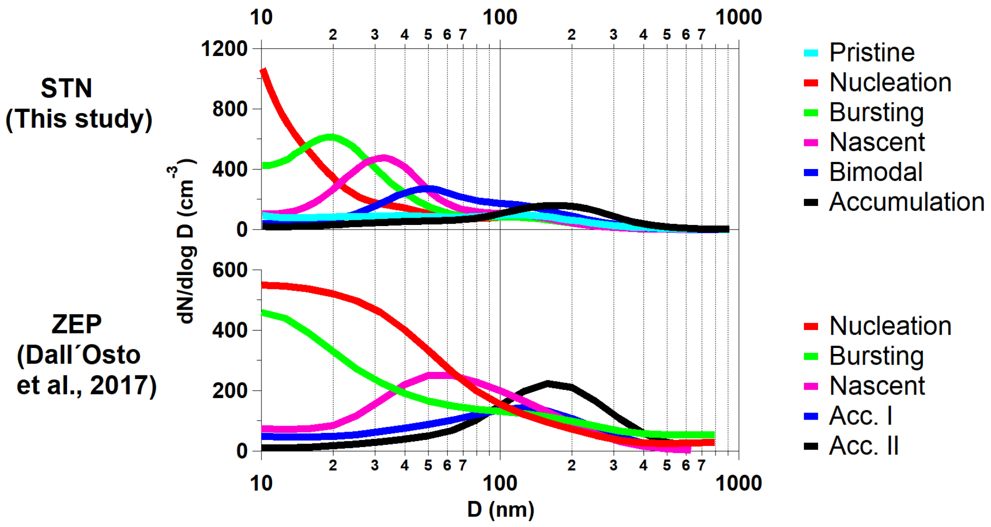

Supplementary Figure S2 Average daily size distributions presented in this study (top, Station Nord - STN) and compared to the one of Dall´Osto et al. 2017 (Ref. 15) at Zeppelin monitoring station (ZEP, bottom).

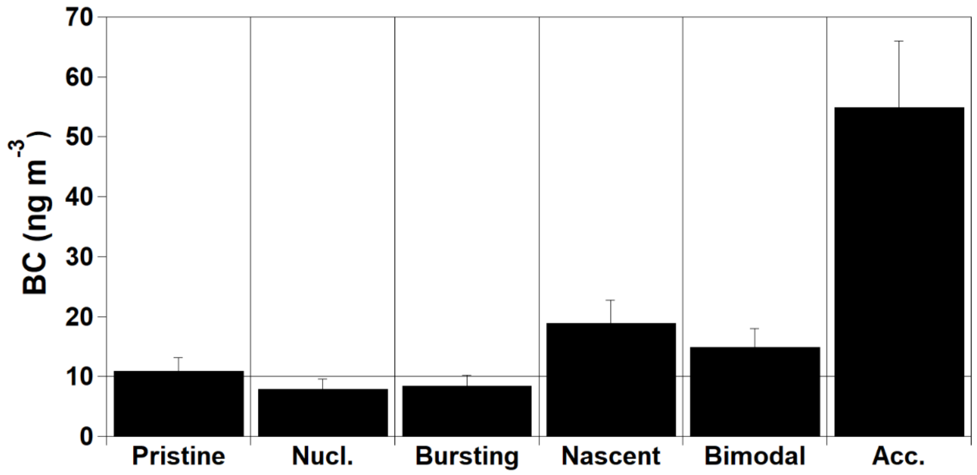

Supplementary Figure S3 Black Carbon concentrations for the six aerosol categories.

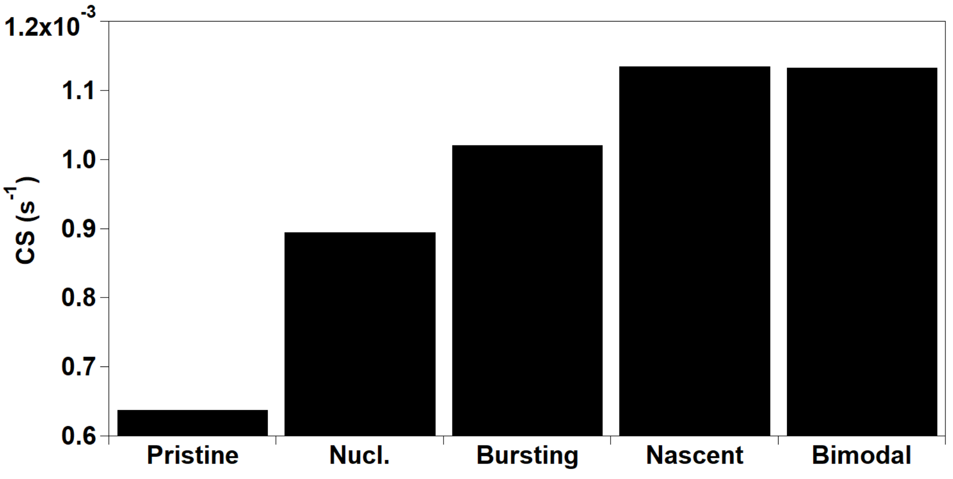

(a)

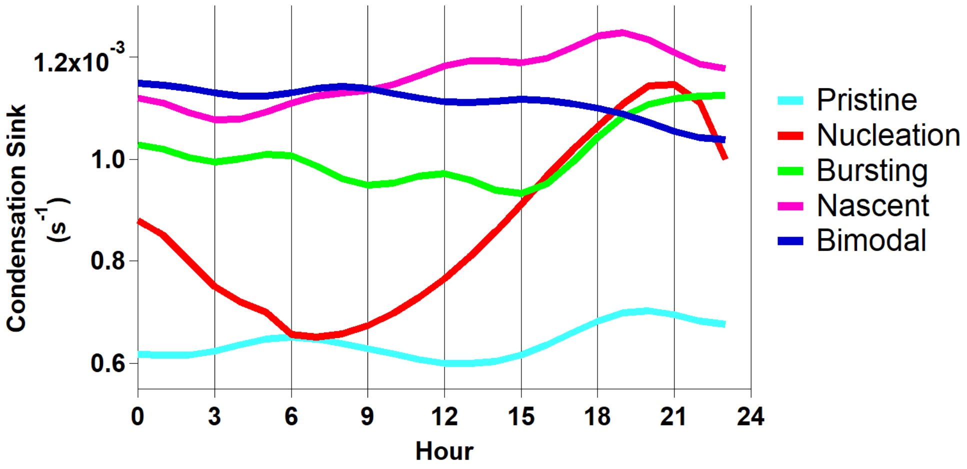

(b) **Supplementary Figure S4** (a) Average daily values and (b) diurnal profile of Condensation Sink for each aerosol category. Please note only the five ultrafine aerosol categories are presented.

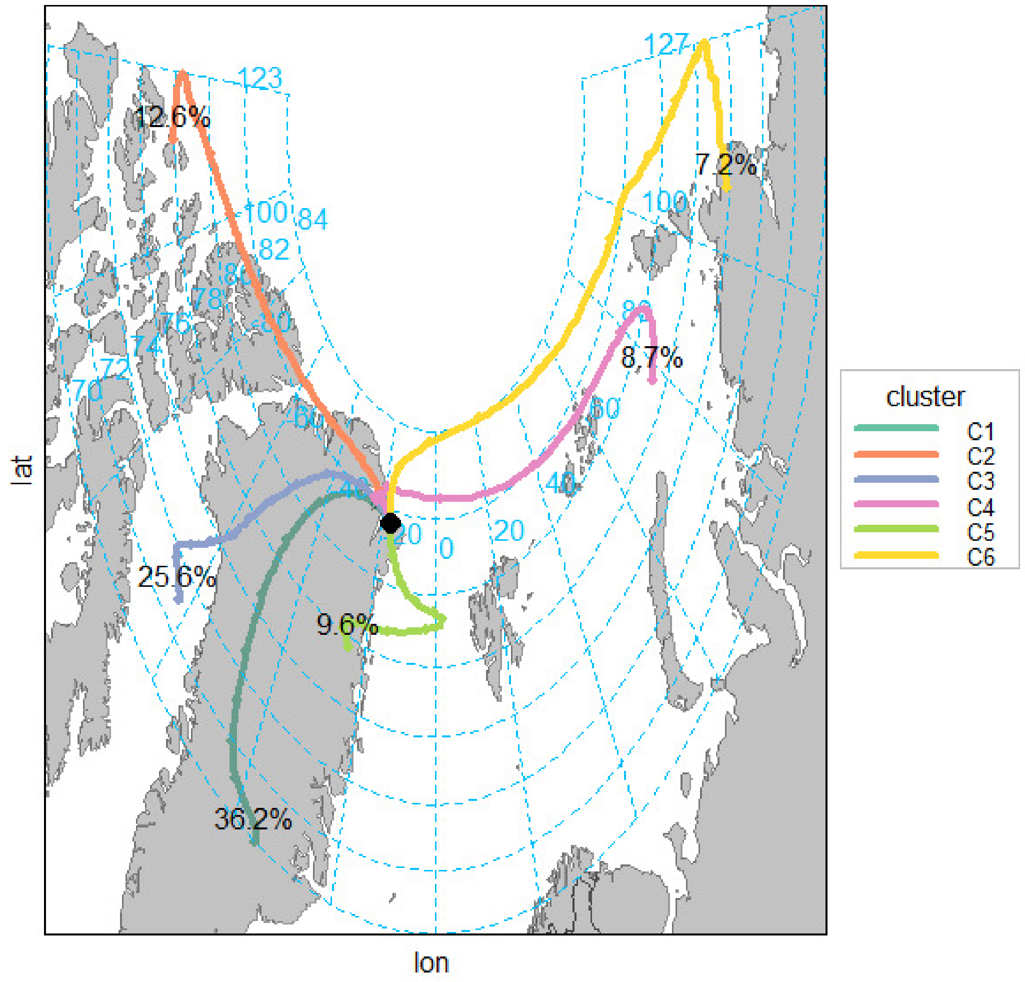

Supplementary Figure S5. Air mass back trajectories cluster analysis. This plot was created using the R software (R Core Team (2016) R: A Language and Environment for Statistical Computing. R Foundation for Statistical Computing, Vienna, Austria. https://www.R-project.org/)

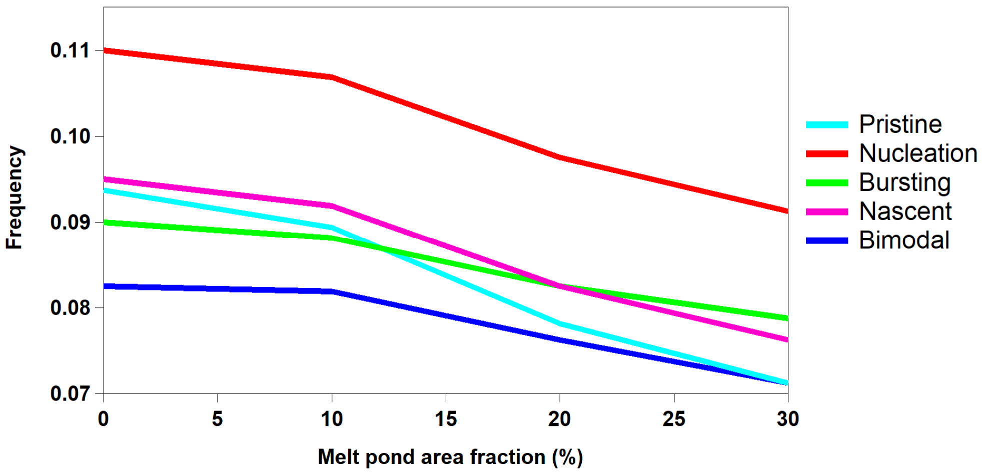

Supplementary Figure S6. Frequency (as fraction of total time) of air mass back trajectories travelling over different melt pond areas for each of the five ultrafine aerosol categories.

Air mass	Pristine	Nucleation	Bursting	Nascent	Bimodal
C 1 (%)	44	25	40	35	40
C 2 (%)	9	15	19	12	14
C 3 (%)	23	26	18	29	24
C 4 (%)	9	10	4	4	4
C 5 (%)	10	13	14	16	18
C 6 (%)	5	10	6	4	1
Total (%)	100	100	100	100	100

Supplementary Table S1. Relation of aerosol SMPS cluster analysis (Figure 1) with air mass back trajectories cluster analysis (cluster 1-6, Figure S5). Percentages for air mass classification are normalized over aerosol cluster category.

Aerosol categories	Geographical regions						
	land	snow on land	sea	sea ice			sum sea + sea ice
				open pack ice	consolidated ice	total	
24h air mass back trajectory analysis							
Pristine (%)	4	52	2	23	19	42	44
**Nucleation** (%)	**6**	**33**	**7**	**31**	**23**	**54**	**61**
Bursting (%)	13	35	4	30	18	48	52
Nascent (%)	13	36	4	29	18	47	51
Bimodal (%)	7	45	4	25	19	44	48
60h air mass back trajectory analysis							
Pristine (%)	3	59	4	19	15	34	38
**Nucleation** (%)	**2**	**37**	**9**	**33**	**19**	**52**	**61**
Bursting (%)	11	40	6	29	14	43	49
Nascent (%)	11	45	5	27	12	40	44
Bimodal (%)	6	54	3	25	12	37	40
120h air mass back trajectory analysis							
Pristine (%)	5	58	4	14	19	33	37
**Nucleation** (%)	**6**	**39**	**12**	**21**	**21**	**43**	**55**
Bursting (%)	9	50	7	15	19	34	41
Nascent (%)	9	52	8	15	16	31	39
Bimodal (%)	6	56	5	15	18	33	38

Supplementary Table S2. Percentages of total time (hours) of air mass back trajectories travelling over different regions (naming ocean, sea ice, snow on land, land), using 1-day, 2.5-day and 5-day air mass back trajectories (24, 60 and 120 hours travelling time previous arriving at STN monitoring site, respectively).
